# The influence of tamoxifen treatment on the oestrogen receptor in metachronous contralateral breast cancer

**DOI:** 10.1038/sj.bjc.6600746

**Published:** 2003-03-04

**Authors:** R Kaas, J L Peterse, A A M Hart, A C Voogd, E J T Rutgers, F E van Leeuwen

**Affiliations:** 1 Department of Surgery, The Netherlands Cancer Institute/Antoni van Leeuwenhoek Ziekenhuis Plesmanlaan 121, 1066CX Amsterdam, The Netherlands; 2 Department of Pathology, The Netherlands Cancer Institute/Antoni van Leeuwenhoek Ziekenhuis Plesmanlaan 121, 1066CX Amsterdam, The Netherlands; 3 Deptartment of Radiotherapy, The Netherlands Cancer Institute/Antoni van Leeuwenhoek Ziekenhuis Plesmanlaan 121, 1066CX Amsterdam, The Netherlands; 4 Cancer Registry, Comprehensive Cancer Centre South, Postbus 231, 5600AE Eindhoven, The Netherlands; 5 Department of Epidemiology, The Netherlands Cancer Institute/Antoni van Leeuwenhoek Ziekenhuis Plesmanlaan 121, 1066CX Amsterdam, The Netherlands

## Abstract

Adjuvant tamoxifen treatment reduces the occurrence of contralateral breast cancer (CBC). The aim of the study was to investigate the hypothesis that adjuvant tamoxifen reduces the occurrence of oestrogen-receptor (ER)-positive CBC, but not the growth of ER-negative CBCs, and to examine survival after diagnosis of CBC. For the study, ER status was immunohistochemically assessed in CBCs of 35 tamoxifen-treated patients and 115 patients without previous hormonal treatment. Cases were retrieved from a series of patients treated from 1984 to 1995 at nine hospitals. The interval between ipsi- and contralateral breast cancer was at least 1 year. It was seen that the proportion of patients with an ER-negative CBC was significantly higher among those with prior tamoxifen treatment: 37% *vs* 18% (*P*=0.047). No difference between the two groups in overall and disease-specific survival following CBC was found. However, the stage differed for both groups: tamoxifen users more often had node-positive contralateral disease (*P*= 0.045). In conclusion, metachronous CBCs developing after 1–3 years of tamoxifen treatment are more often ER-negative breast cancers. So far this does not seem to have a major impact on survival.

Tamoxifen as an antioestrogenic drug was introduced in the seventies and is now widely used as adjuvant and palliative treatment in breast cancer patients. Tamoxifen use as adjuvant treatment prevents new breast cancers or delays disease progression. The drug blocks the binding of oestrogens to the human oestradiol receptors (ER), which are specific proteins in the human breast cell. Oestrogens have, by means of the ER, a proliferating effect on breast cancer cells. The presence of the receptor is a predictor of the endocrine responsiveness of breast cancer cells to tamoxifen (De Placido *et al*, 1990; Early Breast Cancer Trialists' Collaborative Group (EBCTCG), 1998; Chlebowski *et al*, 1999; Radmacher *et al*, 2000).

In the EBCTCG overview of tamoxifen trials, recurrence reductions of 21, 28 and 50%, respectively, after 1, 2 and 5 years of tamoxifen treatment were found in ER-positive breast cancer patients after about 10 years of follow-up. Moreover, adjuvant tamoxifen has an effect on the prevention of contralateral breast cancer (CBC): by the EBCTCG, reductions of 13, 26 and 47%, respectively, were found after 1, 2 and 5 years of tamoxifen treatment (Early Breast Cancer Trialists' Collaborative Group, 1998). These reductions did not depend on dose. Some data suggest that primarily ER-positive CBCs were reduced. In contralateral breast tumours, more often a negative ER status (47% in tamoxifen users *vs* 12% in nonusers) was observed after tamoxifen treatment, but the numbers were very small (Rutqvist *etal*, 1991). A randomised prevention trial comparing tamoxifen (20 mg daily for 5 years) with placebo in healthy women at high risk for breast cancer showed a reduction in the incidence of invasive breast cancer of approximately 50% through 69 months of follow-up (Fisher *etal*, 1998). In the tamoxifen arm, the occurrence of ER-positive tumours was decreased by 69%, but no difference (38 *vs* 31 breast cancers) was seen in the occurrence of ER-negative tumours. The ER+/ER− ratio was 1.07 in tamoxifen users *vs* 4.19 in nonusers. The recent results of the IBIS I trial are consistent with these results; here also no difference in absolute numbers of ER-negative tumours was seen (19 *vs* 19 breast cancers) (IBIS Investigators, 2002).

It is therefore suggested that the benefit of tamoxifen in breast cancer treatment and prevention results from the growth inhibition of occult ER-positive tumours, and that tamoxifen is unlikely to prevent the progression of ER-poor tumours, which is said to be associated with a less favourable prognosis (Fisher *et al*, 1998). In relation to this, and because of other events that influence morbidity, it has been speculated that the preventive effect of tamoxifen on breast cancer mortality in healthy women might be limited.

We examined these issues by comparing the ER status of CBCs between two groups of postmenopausal patients with bilateral breast cancer, who had and had not received adjuvant tamoxifen for their initial breast cancer. In addition, we examined survival in tamoxifen-treated and nontreated patients after the diagnosis of CBC.

## METHODS

For this study, we identified all postmenopausal patients treated for a primary operable invasive breast cancer between 1984 and 1995, who were diagnosed with a CBC at least 1 year later. Patients from two cohorts were selected from the Netherlands Cancer Institute/Antoni van Leeuwenhoek Ziekenhuis (NKI/AvL) in Amsterdam and from the Comprehensive Cancer Centre South (CCCS), which includes patients from eight community hospitals in the southeast of the Netherlands. Follow-up closed in December 1999.

In the study period, approximately 2850 operable postmenopausal women (or 51 years of age and older) were treated for a first primary breast cancer in the NKI/AvL; 1071 received adjuvant tamoxifen. In the hospitals covered by the CCCS, 2923 postmenopausal patients (all stages) were treated in this period; 807 of them received tamoxifen.

At the NKI/AvL, node-positive and node-negative breast cancer patients were randomised in an adjuvant tamoxifen trial to receive tamoxifen 30 mg day^−1^ for 1 or 3 years. ER status was not an eligibility criterion. The standard treatment in the CCCS region was adjuvant tamoxifen at a daily dose of 20 mg. Initially, the use was restricted to patients with ER-positive tumours. In both cohorts, primary node-positive, ER-positive and ER-negative patients were increasingly treated with tamoxifen, 20 mg day^−1^ from 1988 onwards. Treatment for the CBC was only local and no systemic treatment was administered.

The follow-up scheme as advised by the ASCO was the same for the ipsi- as for the contralateral tumour (American Society of Clinical Oncology, 1997).

In total, we identified 150 patients with metachronous CBC: 35 tamoxifen-treated patients and 115 patients without previous tamoxifen treatment. Excluded were patients with ipsilateral local recurrence or distant disease at diagnosis of the CBC, because the differential diagnosis between second primary tumour or metastasis in the breast would be difficult in those patients. Patients with a contralateral ductal *in situ* cancer were included in the analysis, but not patients with lobular *in situ* carcinoma.

The data of the original ER reports (mostly analysis by biochemical methods) were used for the primary breast cancers. The paraffin blocks of the CBCs were requested from the different hospitals for immunohistochemical receptor analysis. Paraffin blocks of 30 patients of the treated group and 98 of the nonusers group were available for receptor analysis (staining with DAKO, clone 1D5, 1 : 500). The ER status was considered positive in cancers with more than 10% nuclear staining.

Medical records were reviewed for clinical data: age, date of diagnosis, stage and ER status of both ipsi- and contralateral tumour, interval between ipsi- and contralateral tumor, tamoxifen treatment with dose and treatment duration and follow-up status up to December 1999.

### Statistical methods

For 2 × 2 tables *P*-values were calculated from Fisher's exact test. The comparison between ipsi- and contralateral breast cancers was done by the test of McNemar.

The triple association between tamoxifen treatment and ER status of first and second tumour was investigated by using binary repeated measurements analysis. Survival curves were calculated using the method of Kaplan and Meier and compared by the log-rank test. For overall survival the event of interest was death from any cause, and for disease-specific survival death from breast cancer.

The statistical package SAS 6, 12 – specifically the procedures TTEST, FREQ, CATMOD, GLM, and LIFETEST – was used for all analyses.

## RESULTS

Among the 150 patients with CBC, 35 (23%) had been treated with adjuvant tamoxifen; 22 from the NKI and 13 from the CCCS. The mean age at first breast cancer was 62.4±7.4 (s.d.) years (range 51 – 77), and the mean age at diagnosis of the CBC was 67.1±7.4 (s.d.) years (range 54–79). The mean age at first breast cancer and CBC did not differ between the tamoxifen- and the nontreated group (see Table 1

**Table 1:** Characteristics of first primary breast cancer in CBC – cases with and without previous tamoxifen treatment

	**Tamoxifen users**		**Tamoxifen nonusers**	
**Characteristics**	***n*=35**	**23%**	***n=115***	**77%**
Mean age at diagnosis±s.d.	62.4±7.4 year		62.9±8.6 year	
Range	(51–77)		(49–81)	
Pathological tumour size				
pT1 (1–20 mm)	9/35	26%	73/115	63%
pT2 (21–50 mm)	20/35	57%	40/115	35%
pT3 (>50 mm)	5/35	14%	2/115	2%
Pathological nodal status				
pN0	6/35	17%	90/115	78%
ER status^a^				
Negative	3/29	10%	12/78	15%
Unknown	*n*=6		*n*=37	
Family history: at least one first degree member	15/33	45%	37/110	34%

BC=breast cancer; ER=oestrogen receptor.

a ER status from original test result, mostly biochemical.

and Table 2

**Table 2:** Characteristics of CBCs in patients with and without previous tamoxifen treatment

	**Tamoxifen users**		**Tamoxifen nonusers**		
	***n*=35**	**23%**	***n*=115**	**77%**	***P*-value**
Mean age at diagnosis±s.d.	67.1±7.4 year		67.1±8.9 year		
Range	(54 – 79)		(51 – 88)		
Interval IBC – CBC					
<2 years	7/35	20%	19/115	17%	
2–5 years	12/35	34%	44/115	38%	
>5 years	16/35	46%	52/115	45%	
Pathol. tumour size					
pTis	5/34	15%	7/112	6%	
pT1 (1–20 mm)	20/34	59%	85/112	78%	0.33^a^
pT2 (21–50 mm)	5/34	15%	17/112	16%	
pT3 (>50 mm)	4/34	12%	3/112	3%	
Pathol. nodal status					
pN0	19/33	58%	87/113	77%	0.045
pN1	14/33	42%	26/113	23%	
ER status^b^					
Negative	11/30	37%	18/98	18%	0.047
Positive	19/30	63%	80/98	82%	
Unknown	*n*=5		*n*=17		

IBC=ipsilateral breast cancer; CBC=contralateral breast cancer; ER=oestrogen receptor;

a pTis+pT1 vs pT2+pT3.

b Immunohistochemical test on paraffin blocks: ER negative ⩽10% of cells containing nuclear staining.

). Of the 5773 patients treated in the two cohorts in the study period, 1878 (33%) received adjuvant tamoxifen. For the tamoxifen-treated patients, a crude CBC frequency of 1.9% was found and for the nonusers, a frequency of 3.0%.

Since tamoxifen was prescribed to node-positive patients in particular, the stage of the primary breast tumour differed between both groups ( Table 1). The tumour stage of the second breast cancer (Table 2) also only slightly differed between the tamoxifen-treated and the nontreated patients, with proportions of pTis+pT1 tumours of 74 and 82%, respectively, (Fisher's exact test *P*=0.33). For nodal stage, a difference between the groups was found: the proportion with pN1 was 42% for the treated and 23% for the nontreated group (*P*=0.045). Overall, relatively more small tumours were found as compared to the first breast cancer, (McNemar's test *P*<0.0001).

Tamoxifen users appear to have more often ER-negative second breast cancers than nonusers: 37 *vs* 18% (*P*=0.047). Among the patients not treated with tamoxifen, the proportions of ER-negative tumours were quite similar for the first and the contralateral breast tumour: 15 and 18%. In the tamoxifen-treated group, however, 10% of the first primary tumours were ER negative, and 37% of the contralateral tumours (McNemar's test *P*=0.016). Therefore, a binary repeated measurement analysis was done, indicating an increase in the proportion of ER-negative tumours between the first and the CBC in tamoxifen-treated patients compared to patients without previous tamoxifen treatment (*P*=0.012). This analysis was carried out on 88 of the patients for whom ER status was known for both tumours.

The difference between the tamoxifen users and nonusers regarding ER negativity of the second breast cancer seems to be especially large Q1in the late (>5 years) CBC group (*P*=0.005). However, this differential effect of interval may still be because of chance (interval tamoxifen interaction: *P*=0.091 in exact logistic regression).

Figure 1

**Figure 1:**
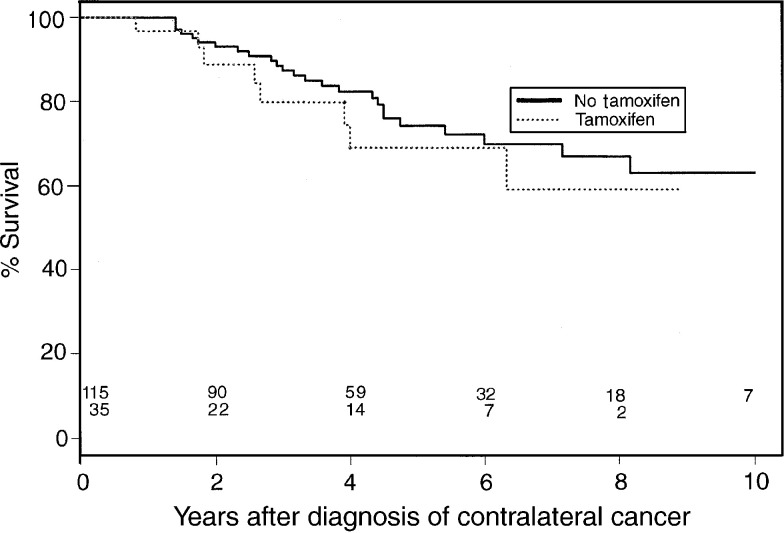
Overall survival curve.

and Figure 2

**Figure 2:**
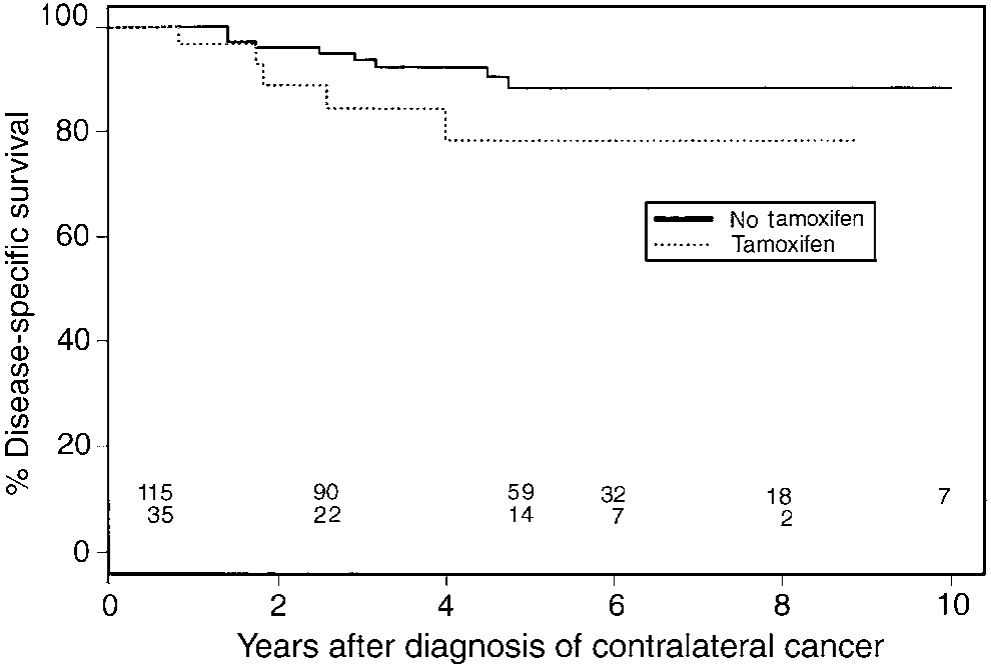
Disease-specific survival curve.

show the overall and disease-specific survival curves from the time of diagnosis of the second breast cancer, according to tamoxifen use for the first breast cancer. Six-year overall survival for nontreated patients was 70 *vs* 69% for tamoxifen-treated patients (*P*=0.45), and disease-specific survival was 88 *vs* 78%, respectively (*P*=0.14). Adjustment for stage did not change these results (Figure 3

**Figure 3:**
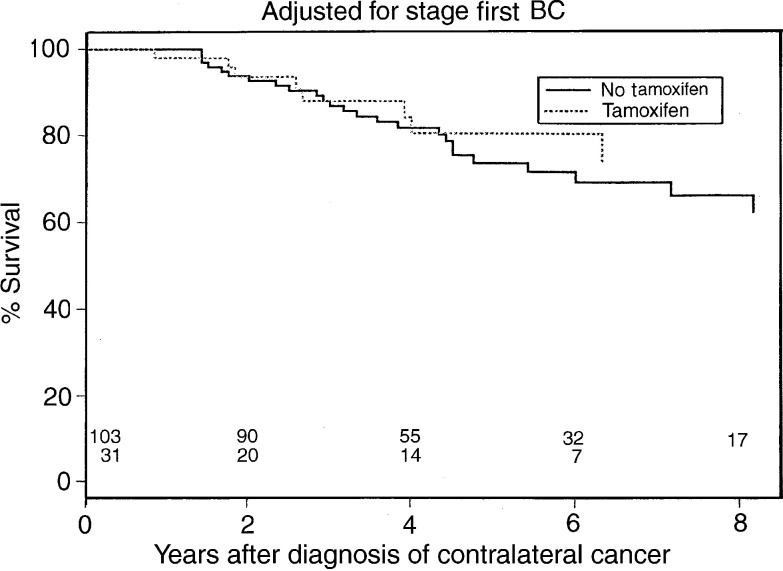
Stage-adjusted survival curve.

).

## DISCUSSION

In this analysis of ER status in CBC patients, we found a greater proportion of ER-negative second cancers in tamoxifen-treated patients than in patients without tamoxifen treatment. The stage of these ER-negative CBCs was more often node positive, but this result did not seem to have a major impact on survival after the diagnosis and treatment of contralateral disease. This finding is in agreement with Rutqvist *et al* (1991) who Q2reported 47% of ER-negative CBCs during and after tamoxifen use *vs* 12% in nonusers. In the recent report of Li *et al* (2001) also an increase was found in ER-negative CBCs after tamoxifen use: 26 *vs* 4% in nonusers. Rutqvist *et al* (1991) did not find differences in tumour size or nodal status in the CBCs of tamoxifen-treated and non treated patients. In the present study, the proportion of early-stage CBC (stage 0+1) was slightly, but not significantly (*P*=0.33), lower in the tamoxifen-treated group (74%) compared to the nontreated group (82%); if a difference at all, sensitivity of mammography (NKI/AvL data not shown) cannot explain this, because sensitivity was 87% in both groups.

BRCA 1 gene mutation carriers have been reported to develop ER-negative tumours more often (Loman *et al*, 1998). In the present study, no identified carriers were present among the tamoxifen users. Only one of three ER-negative patients of the tamoxifen-users group had a weak positive family history. In the nonusers group, two patients were known BRCA 1 mutation carriers; both had negative ER of first and second breast cancer. Thus the greater proportion of second ER-negative breast cancers cannot be explained by a difference in family history.

Radmacher *et al* (2000) found more ER-negative tumours in the second and third year of the tamoxifen treatment. And Rutqvist *et al* (1991) found slightly more ER-negative tumours during tamoxifen treatment or within 3 months after cessation. In the present study, a significant time dependency for ER-negative tumours was not established, but a trend for more ER-negative CBCs was seen 5 years or more since the start of the tamoxifen treatment. This is somewhat paradoxical, assuming that ER-positive tumours, occult at diagnosis of the first breast cancer, are treated by tamoxifen, whereas the ER-negative tumours can grow. In the first 5 years after diagnosis of the first breast cancer, in both treatment groups 21% of ER-negative tumours is seen. But after these 5 years, a difference becomes apparent: of the CBCs in the nonusers, only 10% has a negative ER, whereas 44% of the CBCs in tamoxifen users has a negative ER. This could mean that tamoxifen not only cures but also prevents ER-positive CBCs. This apparent selection of ER-negative breast cancer after tamoxifen use is more surprising as Tarone *etal* (2002) established that the increase in the incidence of breast cancer after 50 years of age is largely because of an increase in ER-positive breast cancers, while the incidence of ER-negative cancers remains constant.

No literature could be found with regard to molecular – biological mechanisms of second primary breast cancers after adjuvant tamoxifen treatment. A review of O'Regan and Jordan (2002) describes the known mechanisms of selective estrogen receptor modulators (SERMs) at a target site, but the proportions of ER coregulator proteins and the pathways have not yet been established. The SERM – ER complex can recruit coactivators and corepressors, and these complexes may modulate genes by different pathways.

In the present analysis, 6-year overall survival after diagnosis of CBC was not different in tamoxifen users and nonusers. A difference in disease-specific survival at 6 years, 88% in nonusers *vs* 78% in tamoxifen users, did not reach statistical significance. Nor was the 6-year overall survival different for both groups after adjustment for the stage of the first tumour. A limitation of these analyses is, however, that the treated and nontreated groups were not totally comparable regarding other tumour characteristics.

In conclusion, the presented results suggest that the CBCs developing after 1–3 years of use of adjuvant tamoxifen are more frequently ER negative, and node positive. This observation, however, does not seem to have a major impact on the overall survival.
